# Are Underweight Shelter Dogs More Likely to Display Food Aggression toward Humans?

**DOI:** 10.3390/ani9121035

**Published:** 2019-11-27

**Authors:** Katherine A. Miller, Emily D. Dolan, Victoria A. Cussen, Pamela J. Reid

**Affiliations:** 1 American Society for the Prevention of Cruelty to Animals, Behavior Rehabilitation Center, Weaverville, NC 28787, USA; 2 American Society for the Prevention of Cruelty to Animals, Strategy and Research, New York, NY 10128, USA; emily.dolan@aspca.org; 3 American Society for the Prevention of Cruelty to Animals, Anti-Cruelty Behavior Team, New York, NY 10128, USA; victoria.cussen@aspca.org (V.A.C.); pam.reid@aspca.org (P.J.R.)

**Keywords:** food aggression, resource guarding, food guarding, dog, behavior, body condition, starvation, cruelty, forensic, legal

## Abstract

**Simple Summary:**

Canine aggression over resources such as food or edible chews is commonly termed food guarding or food aggression. Food aggression directed at humans is relatively common in dogs, including dogs that enter animal shelters. While food aggression is part of dogs’ normal behavior, little is known about why some dogs show food aggression, but others do not. Common animal shelter “lore” suggests that dogs that are underweight or were previously starved are more likely than normal-weight dogs to show aggression toward a human that approaches while the dog is eating. It is assumed that the value of food to the dog is increased by experience with food scarcity, and the value remains elevated even after food is no longer scarce. We tested this common belief by analyzing data from 900 dogs from criminal cruelty cases to look for a relationship between the dogs’ weight and human-directed food aggression. Contrary to common belief, we found that underweight dogs were not more likely than normal-weight dogs to be food aggressive. Based on these results, human-directed food aggression is not a reliable indicator of past food scarcity. This has implications for criminal proceedings, as dogs can have previously experienced starvation without obvious residual behavioral indications in the shelter.

**Abstract:**

It is commonly believed that underweight or emaciated dogs are predisposed to food aggression toward humans. Each year, the American Society for the Prevention of Cruelty to Animals (ASPCA) receives hundreds of dogs from criminal cruelty cases. The dogs range from emaciated to overweight. We analyzed existing data from 900 such dogs to examine the relationship between body condition score and food and chew item aggression toward humans. Across all types of cruelty cases, 9.2% of dogs were aggressive over the food, chew, or both, which is a lower prevalence than that previously reported among shelter dogs. Dogs from cruelty cases originating in New York City were more likely to show aggression over food (z = 3.91, *p* < 0.001) and chew items (z = 2.61, *p* = 0.01) than dogs from large-scale cruelty cases, although it is unclear why. Female dogs were less likely to show food (z = −3.75, *p* < 0.001) and chew item (z = −2.25, *p* = 0.02) aggression compared to males. Underweight dogs were not more likely to display food aggression, but when they did, the aggression was no more severe than that of normal-weight dogs (Fisher’s exact tests = 0.41 and 0.15 for the Food Bowl and Chew Item scenarios, respectively). Breed type was not a significant predictor of aggression. Canine food aggression does not appear to be an aberrant behavior caused by a history of food scarcity but may be related to biological factors such as sex. These findings could prove useful for animal behavior subject matter experts testifying in court or consulting on cruelty cases, as they could speak with scientific validity to the question of whether there is a link between previous food scarcity and the likelihood of food aggression in dogs.

## 1. Introduction

Common animal shelter “lore” suggests that dogs who are underweight or were previously starved are more likely than normal-weight dogs to aggress toward a human that approaches while the dog is eating. It is assumed that the value of food to the dog is increased by experience with food scarcity and remains elevated even after food is no longer scarce. The elevated value of the food presumably motivates the dog to defend it from a potential rival [1,2].

Canine aggression over resources such as food or edible chews is commonly termed food guarding or food aggression. It is generally displayed toward other dogs, humans, or both. This study is focused on food aggression directed at humans, which is relatively common in dogs. About 20% of dogs entering animal shelters display some form of it in a standardized behavior evaluation [3]. While food aggression is part of the normal canine behavioral repertoire, little is known about why some do it while others do not. Many dogs entering animal shelters are underweight, some due to neglect or intentional cruelty, others due to a short-term period of inadequate food (e.g., lost or abandoned dogs who enter shelters as strays), some due to medical reasons (irritable bowel syndrome, cancer), and some due to a combination of these factors. Shelter workers usually do not know much about a dog’s history, but aggressive behavior by underweight dogs, particularly over food, is often assumed to be a result of past negative experiences.

Despite increasing acceptance that food aggression by dogs may be considered normal regardless of cause, many shelters euthanize dogs who display such aggression to people [4,5]. In an animal shelter and in a home, such behavior is a potential risk to human safety. However, it is a behavior that is often situation or environment-specific, responsive to treatment, and readily managed in a home [3,4]. Dogs displaying food aggression are not more likely to be returned by adopters than dogs that are not food aggressive [5]. Better understanding of the causation and prevalence of food aggression across various shelter dog subpopulations may lead to more emphasis on treating and managing the behavior, and fewer dogs being euthanized because of it.

The American Society for the Prevention of Cruelty to Animals (ASPCA) assists the New York City Police Department and various law enforcement agencies around the country to seize and care for animal victims of criminal cruelty. Each year, we receive hundreds of dogs with such origins and administer standardized forensic medical examinations and behavioral evaluations soon after intake. Therefore, the ASPCA has a large, unique dataset allowing analysis of the relationship between dogs’ history, body condition, and food aggression. This study analyzed that dataset with the goals of:

(1) Describing the prevalence and severity of food aggression among canine victims of cruelty or neglect when assessed in a shelter setting,
(2) Examining whether the prevalence and severity of food aggression varies by origin of the dog (i.e., the type of criminal case),
(3) Describing the range of body condition scores found in this population,
(4) Analyzing the relationships between origin of the dog, body condition score, and food aggression,
(5) Examining the odds that an underweight dog will be food aggressive compared to normal or overweight dogs.

These findings could prove especially useful for animal behavior subject matter experts testifying in court or consulting on forensic cases, as they could speak with scientific validity to the question of whether there is a link between previous food scarcity and the likelihood of food aggression in dogs.

## 2. Materials and Methods

### 2.1. Subjects

We examined existing data from 900 dogs entering the care of the ASPCA from January 2012 to July 2016 that originated from criminal cruelty cases. These were cases in which the animals were put into the care of the ASPCA while their legal cases were resolved. These included large and small-scale cases. Larges cases were investigated by local and federal law enforcement agencies in nine different U.S. States, while the small cases were investigated by ASPCA Humane Law Enforcement officers and the New York Police Department (HLE/NYPD). The large-scale cases, involving tens or hundreds of animals, fell into the categories of dogfighting, animal hoarding, substandard dog breeding facilities (puppy mills), and substandard animal sanctuaries/shelters. The smaller scale HLE/NYPD cases, involving individual or small numbers of animals in New York City, NY, included various acts of cruelty or neglect generally directed toward fewer than five animals, with occasional exceptions. These cases included acts of abandonment, beating, burning, stabbing, starvation, and overt criminal acts, but also some cases of small-scale ‘hobby’ dogfighting (as opposed to large-scale professional operations) and hoarding cases.

Sex and breed type were determined by the forensic veterinarian based on visual and physical examination. Age was estimated by veterinary examination based on the eruption of all permanent teeth and the degree of dental wear. Of the 900 total dogs, 91 were juveniles (5 months to 1 year) and 809 were adults (>1 year); 380 were male, 465 were female, and 58 had missing data on sex. Dogs were not differentiated by spay/neuter status for the purposes of this study because there was no a priori hypothesis about this variable. The breed types reported by the examining veterinarians were condensed into two categories: “pit bull type” (n = 530, 58.9%) or “non-pit bull type” (n = 370, 41.1%). “Pit bull type” included dogs labeled as: lab/pit bull mix, pit bull, pit bull mix, and pit mix. All other breed types were coded as “non-pit bull type.” Breed type was included due to the prevalence of pit bull-type dogs in the animals from both large-scale (57%) and small-scale (65%) cases.

### 2.2. Data Collection

Data were collected as part of the ASPCA’s routine documentation of forensic evidence of cruelty.

Medical data, sex, and breed type were recorded during standardized forensic medical exams conducted by veterinarians in the ASPCA’s Forensic Sciences department. Exams were initiated upon the dogs’ arrival at the ASPCA’s shelters where the dogs were housed and cared for until final disposition. Physical examination also included notation of the dog’s body condition score (BCS) on the nine-point Purina Body Condition Scoring System (Appendix A) [6,7]. Dogs scoring 1 to 3 on this scale are considered underweight, those scoring 4 or 5 are considered to be at an ideal weight, and those scoring 6 to 9 are considered overweight.

Behavioral data were collected during standardized evaluations conducted by teams of animal behavior professionals who were Certified Professional Dog Trainers (CPDT) or Certified Applied Animal Behaviorists or Associates (CAAB or ACAAB). The dogs were given at least 3 days after intake to the shelter to settle into their new environments before their behavior was assessed. Dogs who were ill, in pain, debilitated, or in late pregnancy were not behaviorally evaluated until their physical condition stabilized, as determined by the ASPCA’s veterinary medical team. The behavior evaluation was composed of multiple scenarios that simulate what the dog would experience in a typical shelter or home environment, including being petted and handled, being enticed to play, having a bowl of food (‘Food Bowl’ scenario) taken away, having an edible chew item (‘Chew Item’ scenario) taken away, being scolded, meeting a toddler-sized doll, and meeting a friendly dog.

This study examined the data collected during the scenarios in the evaluation in which the dog’s food and then a chew were taken away. In the Food Bowl scenario, the dog was held on a leash by an assistant while the tester gave the dog a paper or metal bowl of food comprised of approximately 1 cup of dry kibble (Iams Adult Small Bites) mixed with approximately 1/3 cup of canned cat food (Friskies Classic Pate’ in Ocean Whitefish and Tuna, Mixed Grill, or Turkey and Giblets flavor). The bowl was placed in front of the dog, and the dog was permitted to engage in eating for a few seconds before the tester approached the dog and, using a plastic hand for safety (Assess-A-Hand^®^, Great Dog Productions, Accord, NY, USA), performed the following four actions with a pause of about 2 s between them: (1) stroked the dog’s back and asked, “Hey, what do you have there?”, (2) touched the dog’s neck and asked, “Hey, what do you have there?”, (3) touched the dog’s muzzle and repeated, “Hey what do you have there?”, (4) firmly pushed the dog’s muzzle out of the bowl and said sternly, “Ok, get out of there!” Each of these actions was performed only when the dog was engaged with the food bowl, sometimes requiring the tester to pause longer than 2 s between actions to allow the dog to resume eating after being touched. Occasionally, a dog would not begin eating when it received the food bowl, or would stop eating and not resume, in which case the tester tried to coax the dog into engaging with it. The dog’s behavior in response to each of the four actions by the tester was scored on a 1 to 3 scale, depending on presence and severity of aggression (Table 1).

After completion of the food bowl scenario, the Chew Item scenario was conducted in the same manner, except that the dog was given a dried pig’s ear or rawhide chew instead of a bowl of food. Scoring was performed in a similar manner (Table 1).

If the dog did not eat at all during the Food Bowl scenario and did not chew on the pig’s ear or rawhide during the Chew Item scenario, their data were not included in this study. If the dog ate or chewed during at least one of the four actions described in the scenarios above, their data were included. For both Food Bowl and Chew Item scenarios, each dog was scored as having shown any aggression or not, and if they did display aggression, the maximum severity of aggression (warning or snap/bite) displayed across all four actions by the tester (or across as many of the four actions as the dog was engaged with the bowl or chew, respectively) was recorded.

### 2.3. Data Analysis

BCS scores were initially combined into three categories for analysis: Underweight, Ideal, and Overweight. However, because only a relatively small proportion of the study population was overweight (see Results), the categories of Ideal and Overweight were combined for statistical analysis (Table 2).

A total of 900 dogs were rated for at least one subtest. Data were recorded during the Food Bowl scenario and the Chew Item scenario for 896 and 641 dogs, respectively. Fewer than 900 dogs were scored in each scenario because some dogs were too fearful to eat or chew, had no interest in the food or chew, or were skipped for a logistical reason such as dietary restrictions.

Frequencies were calculated to determine the prevalence and severity of aggression, and to describe the range of BCS. The severity of aggression displayed over the food and chew was compared between BCS categories using Fisher’s exact test.

Logistic regressions were used to assess the effect of BCS, case type, breed type, sex, and age on the presence of aggression for both the Food Bowl and Chew Item scenarios. The aggression severity categories Warning and Snap/Bite were combined to create a dichotomized outcome variable for the presence/absence of aggression. Covariates were tested for inclusion in the models using a significant likelihood ratio test (*p* < 0.05). All possible interactions were tested for inclusion. The link test and a goodness-of-fit test were used to assess model specification. Sample sizes varied between the two models as follows: logistic regression food scenario n = 841; logistic regression chew scenario n = 599.

All data were analyzed using Stata/IC 13.1 (StataCorp LP, College Station, TX, USA).

## 3. Results

Of the 900 dogs in this study, 422 dogs (47%) were Underweight (BCS of 1 to 3), 418 were Ideal weight (46%), and 60 (7%) were Overweight. The categories of “Ideal” and “Overweight” were combined for analysis; thus, 478 dogs (53%) were Ideal/Overweight (Table 3).

A total of 83 individual dogs (9.2%) were aggressive over the food, chew, or both (see Table 3). Of those, 29 dogs (3.2%) were aggressive over both, while 19 (2.1%) were aggressive over the food only, 26 (2.9%) were aggressive over the chew only, eight (0.9%) were aggressive over the food but had no data on chew, and one (<0.1%) was aggressive over the chew but had no data on the food. Overall, 56 dogs (6.2%) displayed aggression over the food, and 56 (6.2%) displayed aggression over the chew (total does not sum to 83 because 29 of these dogs displayed aggression over both food and chew). Of the 112 acts of aggression observed, about two-thirds were a warning while about one-third were a snap or bite (Table 4).

Results of the Food Bowl and Chew Item scenario logistic models are shown in Table 5 and Table 6, respectively. Controlling for covariates, underweight dogs were not more likely to display aggression over the food or chew item than Ideal/Overweight dogs (z = 0.77, *p* = 0.45 and z = −1.25, *p* = 0.21, respectively). Dogs from the HLE/NYPD cases were 3.6 times as likely to aggress in the Food Bowl scenario (z = 3.91, *p* < 0.001) and 2.4 times as likely to aggress in the Chew Item scenario (z = 2.61, *p* < 0.01) compared to dogs from any other case type. Female dogs were less likely to aggress than male dogs in both the Food Bowl (z = −3.75, *p* < 0.001) and Chew Item (z = −2.25, *p* = 0.02) scenarios. Pit bull-type dogs were no more likely to show aggression than non-pit bull-type dogs in either scenario (Food Bowl z = −0.55, *p* = 0.58; Chew Item z = −0.16, *p* = 0.48). Age was not a predictor of aggression in either the Food Bowl or Chew Item scenario. Among dogs displaying food aggression, there was no association between BCS and severity of aggression (warning versus snap/bite) over the food (Fisher’s exact = 0.41) or chew (Fisher’s exact = 0.15); see Table 4.

## 4. Discussion

Within this population of shelter dogs originating from criminal cruelty cases, underweight dogs were not more likely to display food aggression than ideal/overweight weight dogs during a standardized in-shelter behavior evaluation. Among the dogs that did aggress over food, there was no association between body condition score and severity of aggression. The most food-aggressive dogs displayed warning behaviors (stiffen, growl) and did not escalate to snapping or biting. Dogs originating from the HLE/NYPD cases in New York City were significantly more likely to display food aggression than dogs from other case types. However, compared to previous studies of food aggression in shelter dogs [3,4], the prevalence of food aggression in this study was rather low, even among HLE/NYPD case dogs.

There has been little study of the relationship between aggression and BCS or history of food scarcity in dogs. We know of only one other study that reported body condition score along with food aggression data [8]. However, in that research, only the mean BCS across food aggression categories was reported, and it was in the 4 to 5 range on the 9-point Purina scale (“Ideal” body condition). Thus, apparently few, if any, underweight dogs were included in that study population, and they were not analyzed separately. Nevertheless, their analysis found no association between BCS and food aggression in a standardized shelter behavior evaluation, supporting the present results.

Previous studies of food aggression among the general population of shelter dogs reported that 14.9% to 21% of dogs displayed aggression over a bowl of food, a chew, or both [3,4]. Food aggression is rather common in the pet dog population, too. In the Slovak Republic, 6.2% of survey respondents indicated that their pet dog sometimes, usually, or always displayed aggressive behavior when disturbed while eating versus 11.9% when disturbed while chewing on a treat [9]. In the United States, between 10% [4] and 28.9% [3] of shelter dog adopters reported food aggression in the home after adoption (based on responses to follow-up surveys).

The prevalence of food aggression in this study was notably lower, at 9.2%. Even HLE/NYPD case dogs, which were the most likely to display food aggression in this study, showed a slightly lower prevalence of food (14.4%) and chew (12.7%) guarding than has been previously reported in shelter dogs. While the percentage of pit bull-type dogs in the study population was rather high (59%), this mirrors the prevalence of such dogs in the general shelter population. We found that pit bull-type dogs were not more likely to guard food or a chew.

There are many possible reasons that the prevalence of resource aggression was lower in our samples. It is possible that in-breeding occurred among dogfighting, puppy mill, and hoarding cases, where breeding within the population is common. This may have led to lower genetic and behavioral diversity in those populations. However, even among dogs originating from substandard shelter/sanctuaries, which are presumably the dogs with the most similar histories to the general shelter dog population, the proportion of dogs displaying aggression over the bowl (3.2%) and chew (4.6%) was notably lower than in previous studies. It is also possible that some type of artificial selection occurred that removed more aggressive individuals before shelter intake, e.g., they could have been killed for aggressing against humans. However, this is unlikely to occur in a puppy mill, hoarding situation, or substandard shelter/sanctuary—in which case we would expect a higher prevalence compared to fighting or other cruelty cases. No such pattern emerged in our analyses.

Differences in testing context and or assessment procedures may explain the low overall prevalence of food aggression in the present study compared to prior studies. The context specificity of food aggression has been reported by other researchers. Marder et al. (2013) reported that 45% of dogs who displayed food aggression in a shelter behavior evaluation did not display food aggression in an adoptive home, whereas 22% of dogs who were not food aggressive in the shelter were so, once in a home [3]. Mohan-Gibbons et al. (2012) found that only two of 60 food-aggressive shelter dogs were reported by their adopters to guard food or chews at 3 days, 3 weeks, or 3 months post-adoption [4]. Similarly, the testing methodology in behavior evaluations appears to be an important factor. Bram et al. (2010) reported that even when conducted on the very same dogs, agreement was low among similar behavioral test batteries commonly used to assess non-food-related human- and dog-directed aggression in Switzerland [10].

The higher prevalence of food aggression among the HLE/NYPD case dogs in this study may have been due to some combination of animal, environmental, and experiential differences compared to the dogs originating from other case types. While HLE/NYPD cases include a large percentage of pit bull-type dogs, pit bull types were not more likely to show aggression compared to non-pit bull types. Most HLE/NYPD dogs had been in a home, basement, or backyard at some point in their lives, presumably interacting with people fairly regularly, which is likely not true of most dogfighting or puppy mill case dogs. However, dogs from hoarding and sanctuary origins would likely have lived in a home or backyard at some point in their histories, and dogs from all case types experienced neglect and cruelty from humans, so HLE/NYPD dogs were not unique in these respects. However, HLE/NYPD dogs were more likely to come from situations in which there were only a small number of dogs, as compared to the other types of cases, where large numbers of dogs were often living in close proximity.

There were also two procedural differences in the behavioral evaluation of the HLE/NYPD dogs compared to other case types. First, due to logistical reasons, the chew that the HLE/NYPD dogs were tested with was a rawhide, while the chew used for all other case types was a dried pig’s ear. Dogs may have perceived the rawhide as a more valuable food item compared to the pig’s ear; however, this does not explain the higher incidence of food aggression over the food, which was the same across all case types.

Second, HLE/NYPD dogs were usually taken to the evaluation room at least once before the standardized behavior evaluation to engage informally with people and for exercise and enrichment. Thus, the dogs were somewhat familiar with the room and the testers before the evaluation as administered. For logistical reasons, this was not possible for dogs from other case types. Therefore, the HLE/NYPD dogs may have been more comfortable in the testing environment and their behavior in the evaluation may have been affected by this increased familiarity.

Among the dogs that did display food aggression in this study, warning (stiffening, growling) was more common than biting, and only 2.7% of the dogs in this population snapped or bit over the food or chew. This is notable, especially considering that this assessment involved pushing on the dog’s face while it had possession of the food item, even if the dog was already growling in warning. Most shelter workers and pet owners likely desist in efforts to gain access to a food item when a dog growls at them. Much dog aggression is ritualized and serves to settle conflict without inflicting injury. Marder et al. (2013) found that most adopters of food aggressive dogs did not consider the behavior a challenge to keeping the dog as a pet [3]. Therefore, instead of euthanizing shelter dogs who display warning behaviors over food or chews, educating staff and adopters to recognize and heed such warnings may be sufficient to minimize the risk of food-related bites.

The findings that past food scarcity was not associated with food aggression and that dogs from cruelty cases were not more food aggressive than “average” shelter dogs as reported in other studies e.g., [3], indicate that food aggression is not an aberrant behavior resulting from a stressful life experience. Furthermore, if life experience ‘caused’ food aggression, adult dogs would be predicted to show more aggression than juvenile dogs, as they would have more time to experience food scarcity. We did not find a significant effect of age, supporting the view that food aggression in a normally occurring canine behavior that is exhibited by a small percentage of the population regardless of life history.

The realization that food aggression is not linked to a history of food scarcity is potentially relevant to court testimony in cruelty cases. A defense attorney could claim that the lack of food aggression in a behavior evaluation is evidence that a dog did not suffer from inadequate food resources. The findings provided by this research should be sufficient to refute this argument in a court of law.

## 5. Conclusions

Underweight dogs were not more likely to aggress over food, nor to display more severe food aggression. Overall, in this population of dogs from criminal cruelty cases, food aggression was somewhat less prevalent than in the general shelter dog population, as reported by other researchers. Furthermore, most food-aggressive dogs did not escalate beyond stiffening and growling, even though their face was physically pushed away from the food items. Therefore, the risk of bites due to food aggression in this population may be considered relatively low and was not predictable based on body condition. Food aggression does not appear to be an aberrant behavior caused by a history of food scarcity or cruelty and is likely within the normal range of behavior in dogs. Furthermore, a lack of food aggression does not speak to the question of whether the dog suffered from inadequate nutrition previously.

## Figures and Tables

**Table 1:** Scoring system for Food Bowl and Chew Item scenarios of the behavior evaluation. Each dog was scored when the tester performed each of four actions: Stroke the dog’s back, touch the dog’s neck, touch the dog’s muzzle, and push the dog’s muzzle. The maximum score for each dog was used for data analysis.

**No aggression = 1**
Remains relaxed. Eats (chews) at the same pace, looks at tester or hand and/or moves away from bowl (chew).
Leaves bowl (chew) to interact with tester. May lick or playfully nip at the Assess-a-Hand or tester.
Eats (chews) but seems nervous. Crouched posture, tail down/tucked. Might watch people in room while eating. Might flinch, turn to look at hand or stop eating (chewing) when touched. No guarding behavior.
Motivated to eat (chew)—not stiff/rigid. Resists being pushed out of bowl (away from chew), hunkers over it, body blocks, picks it up and moves away and/or eats (chews) more quickly but does not escalate. No aggression.
**Warning = 2**
Becomes stiff/rigid—no escalation. Gives hard stare, freezes, hunkers down over bowl (chew), body blocks, and/or eats (chews) more quickly but does not escalate when pushed.
Becomes stiff/rigid—threatens. Lifts lip or growls, may raise hackles and/or give “hard eye”.
**Snap/bite = 3**
Becomes stiff/rigid—bites. Tries to bite or bites the Assess-a-Hand. May chase after tester.

**Table 2:** Nine-point scoring system used by veterinarians to indicate a dog’s body condition. Based on the Nestlé Purina Body Condition System [6,7] (Appendix A).

**Underweight**	
1	Ribs, lumbar vertebrae, pelvic bones, and all bony prominences evident from a distance. No discernible body fat. Obvious loss of muscle mass.
2	Ribs, lumbar vertebrae, and pelvic bones easily visible. No palpable fat. Some evidence of other bony prominence. Minimal loss of muscle mass.
3	Ribs easily palpated and may be visible with no palpable fat. Tops of lumbar vertebrae visible. Pelvic bones becoming prominent. Obvious waist and abdominal tuck.
**Ideal/Overweight**	
4	Ribs easily palpable, with minimal fat covering. Waist easily noted, viewed from above. Abdominal tuck evident.
5	Ribs palpable without excess fat covering. Waist observed behind ribs when viewed from above. Abdomen tucked up when viewed from side.
6	Ribs palpable with slight excess fat covering. Waist is discernible viewed from above but is not prominent. Abdominal tuck apparent.
7	Ribs palpable with difficulty; heavy fat cover. Noticeable fat deposits over lumbar area and base of tail. Waist absent or barely visible. Abdominal tuck may be present.
8	Ribs not palpable under very heavy fat cover, or palpable only with significant pressure. Heavy fat deposits over lumbar area and base of tail. Waist absent. No abdominal tuck. Obvious abdominal distention may be present.
9	Massive fat deposits over thorax, spine, and base of tail. Waist and abdominal tuck absent. Fat deposits on neck and limbs. Obvious abdominal distention.

**Table 3:** Frequencies of body condition score (BCS) and food guarding by case type. Numbers in parentheses are the percent of the total in that row. HLE/NYPD: New York Police Department.

Type of Case	Number of Dogs	Underweight BCS = 1 to 3	Ideal/Overweight BCS = 4 to 9	Aggressive over Food *	Aggressive over Chew *
Dog fighting	327	175 (53.5)	152 (46.5)	13 (4.0)	16 (4.9)
HLE/NYPD	229	161 (70.3)	68 (29.7)	33 (14.4)	29 (12.7)
Hoarding	12	0 (0)	12 (100)	0 (0)	0 (0)
Puppy mill	113	32 (28.3)	81 (71.7)	3 (2.7)	1 (0.9)
Substandard shelter/sanctuary	219	54 (24.7)	165 (75.3)	7 (3.2)	10 (4.6)
Total in study	900	422 (46.9)	478 (53.1)	56 (6.2.)	56 (6.2)

* Dogs were considered aggressive if they gave a warning, snapped, or attempted to bite.

**Table 4:** Maximum severity of aggression observed over the food bowl and chew by body condition score category. Data in parentheses are the percent of the total in that row and may not sum to 100% due to rounding.

**Food**				
**Body Condition**	**No Aggression**	**Warning**	**Snap/Bite**	**Total**
Underweight	387 (91.9)	17 (4.0)	16 (3.8)	421
Ideal/Overweight	453 (95.4)	15 (3.2)	8 (1.7)	475
Total	840 (93.8)	32 (3.6)	24 (2.7)	896
**Chew**				
**Body Condition**	**No Aggression**	**Warning**	**Snap/Bite**	**Total**
Underweight	304 (91.8)	16 (4.8)	11 (3.3)	331
Ideal/Overweight	281 (90.6)	23 (7.4)	6 (1.9)	310
Total	585 (91.3)	39 (6.1)	17 (2.7)	641

**Table 5:** Results from a logistic regression for the presence or absence of aggression in the Food Bowl scenario.

Case Type	Odds Ratio	Std. Err.	z	*p* > |z|	95% Conf. Interval	
BCS, Underweight	1.29	0.4	0.8	0.45	0.67	2.49
Case Type, HLE/NYPD	3.59	1.2	3.91	0.00	1.89	6.81
Breed Type, Pit Bull	0.84	0.3	−0.55	0.58	0.44	1.58
Sex, Female	0.26	0.1	−3.75	0.00	0.13	0.53
Age, Adult/Senior	1.67	1.0	0.82	0.41	0.49	5.66

**Table 6:** Results from a logistic regression for the presence or absence of aggression in the Chew Item scenario.

Case Type	Odds Ratio	Std. Err.	z	*p* > |z|	95% Conf. Interval	
BCS, Underweight	0.65	0.2	−1.25	0.21	0.33	1.28
Case Type, HLE/NYPD	2.44	0.8	2.61	0.01	1.25	4.78
Breed Type, Pit Bull	0.95	0.3	−0.16	0.87	0.48	1.85
Sex, Female	0.49	0.2	−2.25	0.02	0.26	0.91
Age, Adult/Senior	3.53	2.6	1.70	0.09	0.82	15.09

## References

[1] Maier R. (1998). Comparative Animal Behavior: An Evolutionary and Ecological Approach.

[2] Scott J.P., Fuller J. (1965). Genetics and the Social Behavior of the Dog.

[3] Marder A.R., Shabelansky A., Patronek G.J., Dowling-Guyer S., D’Arpino S.S. (2013). Food-related aggression in shelter dogs: A comparison of behavior identified by a behavior evaluation in the shelter and owner reports after adoption. Appl. Anim. Behav. Sci..

[4] Mohan-Gibbons H., Weiss E., Slater M. (2012). Preliminary investigation of food guarding behavior in shelter dogs in the United States. Animals.

[5] Mohan-Gibbons H., Dolan E.D., Reid P., Slater M.R., Mulligan H., Wise E. (2018). The Impact of Excluding Food Guarding from a Standardized Behavioral Canine Assessment in Animal Shelters. Animals.

[6] Mawby D., Bartges J.W., Moyers T., Cottrell T., Davignon A., Laflamme D.P. (2001). Comparison of body fat estimates by dual-energy x-ray absorptiometry and deuterium oxide dilution in client-owned dogs. Comput. Cont. Educ. Pract..

[7] Laflamme D. (1997). Development and validation of a body condition score system for dogs. Canine Pract..

[8] Lyle J., Kapla S., da Silva S.P., Maxwell M.E. (2017). Persistence of food guarding across conditions of free and scheduled feeding in shelter dogs. Appl. Anim. Behav. Sci..

[9] Matos R.E., Jakuba T., Mino I., Fejsakova M., Demeova A., Kottferova J. (2015). Characteristics and risk factors of dog aggression in the Slovak Republic. Vet. Med.-Czech.

[10] Bräm M., Doherr M., Lehmann D., Mills D., Steiger A. (2008). Evaluating aggressive behavior in dogs: A comparison of 3 tests. J. Vet. Behav..

